# Facial Neuralgia

**Published:** 1883-08

**Authors:** 


### FACIAL NEURALGIA.

*Facial neuralgia, originating in the left supra-orbital nerve, and finally affecting other branches, relieved by section of the infra-orbital nerve.*

Mrs. M., aged 68, had for some years suffered with pain, which arose first in the left supra-orbital nerve, and now for some month affected with equal and extreme severity the infra-orbital nerve, and at times the infra-maxillary.

As every imaginable drug had been used, and galvanics failed to relieve, I decided to divide both the supra and infra-orbital nerves at one sitting.

The case is brought forward to illustrate a point of practical value, and of extreme importance. If I had had to choose which single nerve I should sever, I should certainly have selected the supra-orbital, in which the pain began. It chanced that I was indisposed at the time set for the operation, and therefore sent word to my colleagues, Dr. Hunt, who operated, and Dr. Morton, who assisted him, to go on without me. In this consultation they concluded as a measure of prudence to divide but one nerve, and not being aware of the reasons which would have then led me to choose the supra-orbital, they divided the infra-orbital nerve.

The results were, however, to annihilate pain in all branches of the fifth nerve, and to leave on my mind a most valuable lesson, since nine months later the same satisfactory condition of things still exists.

The mode of reaching this nerve is not a matter of indifference. In this case the antrum was broken into, and, if I correctly remember, my friend, Prof. Brinton, who has operated for me several times on the infra-orbital, prefers this operation. On the whole, however, it seems to me desirable not to break into the antrum. Indeed, I should like in a future case merely to cut the nerve in front, and again far back in the orbit, and then to leave a small plug of bone or ivory in the little canal, or to close the canal with dental cement. I do not observe that in this case the scar is tender, nor in fact is it apt to be—whilst it is frequently the case when incisions are made on the forearm and a large nerve is cut, that the cicatrix remains tender.

*Neuralgia of left inferior maxillary nerve; extension of pain to other branches; Section of nerve; Return of pain; Second section and obliteration of canal with dental cement.*

January 28, 1883. Miss ——, of New Jersey, aged 43, underwent in April, 1881, by my advice, a resection of the inferior dental nerve on the left side, the operation being performed by Dr. Morton. The case and the immediate results were reported in the *Medical News* for March 11, 1883.

After a long period of ease, some time in March or April, 1882, the pain returned in the jaw at the old seat, and in June, 1882, had become as bad as before. The pain had all the usual peculiarities of neuralgia, and was not limited to the lower nerve, but was felt in both the temporal and orbital branches. At my desire, her home adviser, Dr. Ed. North, of Hammonton, gave her very large doses of aconite, which certainly abolished the pain; but in December, 1882, it returned anew, and in January, 1883, she was re-admitted in a pitiable state of suffering.

On close study it was found that sensation had been restored in the area figured in my last report of her case as having lost it. In some places the touch sense was still imperfect, but it was nowhere destroyed, and throughout a needle prick could be felt.

Clearly the nerve had been re-made, and I was again face to face with this difficult question. After exhausting all means at our disposal, it was agreed in consultation, to seek for the nerve in the canal at the point where formerly it had been severed, and to divide it anew.

At the same time I felt that the operation might fail, like the last one, to give permanent relief, but that at least I should be more secure if I could in some way provide against re-union of the nerve ends.

I had thought of plugging the canal with bone or ivory, or of thrusting periosteum into it, but finally decided to fill it with dental cement. On January 28th, Dr. Morton operated, the patient having been etherized.

On exposing the bone, a small trephine was applied about an inch and a quarter in advance of the angle of the jaw, but the canal thus uncovered was ill-defined, and amidst the crushing caused by the trephine the bleeding and the obscure cancellous structure, we could find or see no re-made nerve. When the tissues were pushed back a little the old trephine mark was disclosed. It had filled up with bone except for one opening about a line wide, from which projected a button-like prominence, which proved to be a stump of nerve tissue. Unhappily the knife had swept over it, and whether or not it furnished filaments running forward over the bone cannot now be known.

On trephining so as to include it, we failed again to trace filaments running along the irregular canal, which certainly existed. The operation enabled us to pull out the nerve trunk some distance, and after stretching to sever it. A more careful search was then made for the filaments presumed to have re-connected the central end with the sensitive skin spaces on the chin. Finding none, the canal was cleaned out, and the two ends of the canal thoroughly filled with warm dental cement, which admits of being easily molded when hot, and then hardens.

What is to be the result of this very novel procedure we have yet to see. Sensation was again destroyed in the region fed by the inferior maxillary nerve, showing that the nerve had been re-made, and again severed during the operation. At this date—May 10, 1883—there has been a recent return of neuralgic pain, but so far no inflammatory disturbance from the presence of the cement, as to the use of which both Dr. Morton and I have had such anxiety, as naturally attends the use of perfectly new methods. The same mode of obliterating the canal has been more recently resorted to by Dr. Morton in another case of resection of the infra-maxillary nerve.

